# An Important Epoch in Medical Education

**Published:** 1914-11-15

**Authors:** 


### AN IMPORTANT EPOCH IN MEDICAL EDUCATION.

The opening of the session of 1914-1915 in the medical schools marks an important epoch in medical education in this country. It is noteworthy that this fall thirty-seven medical colleges, for the first time, are requiring for admission one year of collegiate work in addition to a four-year high school education. This standard, which in 1904 was considered as "ideal," has now become the essential minimum and has been adopted by eighty-four of the 101 medical colleges, and by twenty-two of the forty-nine state licensing boards. In addition to this, thirty-four of these colleges and seven of the state licensing boards have gone further and adopted the requirement of two years of collegiate work as the minimum preliminary standard. This is indeed a marvelous improvement over conditions which existed only a brief ten years ago, when of the 166 medical colleges only four were requiring any collegiate work for admission; and comparatively few were requiring even a completed four-year high school education. Entrance requirements, meanwhile, may be taken as an index of the other remarkable improvements made in medical education during the last decade. The general adoption of the higher entrance standard has resulted in a remarkably increased attendance in colleges of arts and sciences. In fact, some of these institutions which formerly had small enrolments now find their classes so increased that provision for a larger equipment and additional teachers has been necessary. Again, the general adoption of this higher standard makes the entrance requirements of all medical colleges more uniform. As a consequence, students are exercising a larger freedom in selecting the institution they are to attend and will usually select the better one. This is shown to be a fact, since colleges which have for several years had small enrolments on account of their higher entrance standards this year report marked increases in the numbers admitted. More important, however, is the fact that the great majority of students from now on will be better qualified to enter on the study of medicine, and to master the greater complexities of the modern medical curriculum. Finally, in the success of the campaign for higher entrance standards the public has the assurance that the medical schools hereafter will turn out a larger proportion of thoroughly qualified physicians.—*Journal A. M. A.*

